# Do erythrocytes subjected to cardiopulmonary bypass exhibit changes in their membrane mechanical properties?

**DOI:** 10.1186/cc11950

**Published:** 2013-03-19

**Authors:** T Clark, S Jewell, M Sair, P Petrov, P Winlove

**Affiliations:** 1 Derriford Hospital, Plymouth, UK; 2 University of Exeter, UK

## Introduction

Whole blood experiments suggest that cardiopulmonary bypass (CPB) causes red blood cell (RBC) trauma and changes in deformability that may contribute to postoperative microcirculatory dysfunction. We used a novel fluctuation microscopy technique to quantify the effects of CPB on RBC elasticity at a cellular level.

## Methods

We collected blood samples from elective cardiac surgery patients pre (at induction) and post (immediately, each day until CICU discharge) CPB. Thermal fluctuations of individual RBCs were recorded using a high-frame-rate camera allowing a complete analysis of RBC shape variation over time. Mean elasticity of the cell membrane was then quantified for each sample collected.

## Results

Fifteen patients were recruited. Table 1 displays the results. RBC thermal fluctuation is measured relative to pre-bypass values. An increase in RBC fluctuation marks a decrease in stiffness. CPB caused two distinct changes in RBC elasticity; pre fix A indicates samples where stiffness increases or shows no change, B those where stiffness decreases. Data on day 2 were not collected in patients discharged from the CICU. CPB type or time had no apparent impact on RBC response to CPB.

**Table 1:** Change in RBC thermal fluctuation relative to baseline: two distinct groups seen

Patient	A1	A2	A3	A4	A5	A6	A7	B8	B9	B10	B11	B12	B13	B14	B15
Post CPB	+0.1	-0.4	-0.1	-0.5	-0.1	-0.2	-0.1	+0.5	0	+0.4	+0.1	+0.2	0	+0.2	+0.1
Day 1	0	0	+0.1	-0.1	-0.1	+0.1	+0.1	0	+0.5	+0.4	+0.7	+0.4	+0.6	+0.5	+0.7
Day 2	-0.2	+0.1	0	NA	NA	0	NA	+0.5	-0.1	+0.4	NA	+0.1	NA	NA	NA

## Conclusion

RBC thermal fluctuation analysis quanties the impact of CPB on erythrocyte membrane elasticity. We clearly identified two separate RBC elasticity responses to CPB. This finding is contrary to traditional flow measurement techniques that suggest CPB impairs whole blood flow and reduces RBC deformability.
